# Novel Epigenetic Techniques Provided by the CRISPR/Cas9 System

**DOI:** 10.1155/2018/7834175

**Published:** 2018-07-08

**Authors:** Nina Xie, Yafang Zhou, Qiying Sun, Beisha Tang

**Affiliations:** ^1^Department of Geriatric Neurology, Xiangya Hospital, Central South University, Changsha, Hunan 410008, China; ^2^National Clinical Research Center for Geriatric Disorders, Central South University, Changsha, Hunan 410078, China; ^3^Department of Neurology, Xiangya Hospital, Central South University, Changsha, Hunan 410008, China

## Abstract

Epigenetics classically refers to the inheritable changes of hereditary information without perturbing DNA sequences. Understanding mechanisms of how epigenetic factors contribute to inheritable phenotype changes and cell identity will pave the way for us to understand diverse biological processes. In recent years, the emergence of CRISPR/Cas9 technology has provided us with new routes to the epigenetic field. In this review, novel epigenetic techniques utilizing the CRISPR/Cas9 system are the main contents to be discussed, including epigenome editing, temporal and spatial control of epigenetic effectors, noncoding RNA manipulation, chromatin in vivo imaging, and epigenetic element screening.

## 1. Introduction

Epigenetics classically refers to the inheritable changes of hereditary information without perturbing DNA sequences. DNA methylation, demethylation, hydroxyl-methylation, histone modification, chromatin remodeling, gene imprinting, and noncoding RNA are the central mechanisms involved. They play important roles in diverse biological processes including gene regulation, iPSC reprogramming and maintenance, genomic imprinting, X-chromosome inactivation, aging, neurodegeneration, autoimmune modulation, and tumorigenesis [1–4].

Adapted from a natural immune defense system in bacteria, the clustered regularly interspaced short palindromic repeat- (CRISPR-) associated protein 9 (Cas9) system, abbreviated as the CRISPR/Cas9 system, is a site-specific genome editing tool that could be implemented to target and mutate specific genomic regions in eukaryotic cells, especially in mammalian cells [5]. The rationale is described below: the in vivo CRISPR/Cas9 system comprises two core components, a Cas9 nuclease and a guide RNA sequence. The guide RNA is programmable. By changing the sequences of guide RNA, researchers could target Cas9 nuclease to almost any locus in the genome precisely. After being delivered into the cell of interest, guide RNA will direct Cas9 nuclease to the target via complementary matching with the corresponding genomic DNA sequence. Flanking by a NGG protospacer adjacent motif (PAM) is a prerequisite for a piece of DNA sequence to be a qualified target. Once guide RNA finds its target, the Cas9 nuclease will transit from the binding state to the cutting state with the help of PAM, subsequently generating a double-stranded break (DSB). Depending on the presence of a repair template, the DSB will be rejoined either by the nonhomologous end joining (NHEJ) or the homology-directed repair (HDR) mechanism. The former is more error-prone, while the latter is more precise [6–11]. Briefly, by designing specific guide RNA sequence and inducing appropriate downstream repair mechanism, researchers can utilize this method to achieve genome modifications flexibly.

Notably, since the emergence of CRISPR/Cas9 technology, diverse applications have been explored beyond genome editing. Here, we will focus on the new toolkit that CRISPR/Cas9 has provided to us for epigenetic research.

### 1.1. Epigenome Editing

Epigenome editing refers to the targeted rewriting of epigenetic markers [1, 12]. On the one hand, it could be used to selectively modify epigenetic marks at a given locus to explore mechanisms of how targeted epigenetic alterations would affect transcription regulation and cause subsequent phenotype changes. For example, it has been reported that inducing histone methylation or acetylation at the *Fosb* locus in the mice brain reward region, nucleus accumbens, could affect relevant transcription network and thus control behavioral responses evoked by drug and stress [13–15]. On the other hand, epigenome editing has the potential for epigenetic treatment, especially for the disorders with abnormal gene imprinting or epigenetic marks. Targeted epigenetic silencing or reactivation of the mutant allele could be a potential therapeutic approach for diseases such as Rett syndrome and Huntington's disease [12, 16–19]. DNA-binding protein domains, such as zinc finger (ZFN) or transcription activator-like effector nuclease (TALEN), fused with transactivators have been demonstrated to be feasible methods for epigenome editing; however, the protein synthesis step is costly and labor-consuming, which makes it difficult for such methods to be widely used, for the binding specificity of zinc finger and TALEN is determined by the amino acid sequences within their repeat domains. Changing genomic targets means changing amino acid sequences [20–23]. On the contrary, the CRISPR/Cas9 method described above overcomes this shortcoming due to its cost-effectiveness and easy-manipulating. Instead of redesigning amino acid sequences and synthesizing new DNA-binding proteins, what we need to do in the CRISPR/Cas9 system is redesign the programmable guide RNA sequences and synthesize a new expression cassette. Thanks to the advent of the novel CRISPR/Cas9 technology, the field of epigenome editing begins to thrive.

To achieve CRISPR/Cas9-mediated epigenome editing, the main strategy is fusing the Cas9 protein with a transcription repressor or activator domain, which was known as an epigenetic effector (epieffector) [21]. To be specific, the adaption is inactivating the Cas9 nuclease first and further fusing it with an epieffector domain. The deactivated (dCas9) has no nuclease activity but functions as a DNA-binding domain. Accumulating evidence has proved that this dCas9-epieffector fusion complex is an efficient epigenome editing tool.

For example, when the fused epigenetic effector domain was Krüppel-associated box (KRAB), using the dCas9-KRAB complex to target and induce locus-specific deposition of H3K9me3 at the *HS2* enhancer region, researchers successfully silenced multiple globin genes in K562 cells. Off-target perturbation in global gene expression seemed not to be an issue in this study [24].

When the fused domain was LSD1, using the dCas9-LSD1 complex to target *Tbx3*, a gene implicated in the maintenance of pluripotency, researchers observed downregulation of *Tbx3*, loss of H3K27Ac at the enhancer, and impaired pluripotency in embryonic stem cells. In addition, this study compared specificity for enhancer targeting between dCas9-LSD1 and dCas9-KRAB complexes. Interestingly, it was concluded that dCas9-LSD1 was enhancer-specific; however, the specificity of the dCas9-KRAB complex was questionable, for it may actually be silencing the promoter or changing the chromatin structure instead of targeting the enhancer to achieve *Tbx3* downregulation. The difference in the experimental methods, such as origins of Cas9 nuclease, cell types of interest, and genes to be manipulated, might account for such contradictory findings [25].

When the fused domain was p300 core, which was an acetyltransferase, using this dCas9-p300 core complex, researchers activated the *Myod* gene at a regulatory region distal to the promoter, upregulated the *Oct4* gene from a regulatory region proximal to the promoter, and induced transcription in three-fourths of downstream hemoglobin genes by targeting several DNase hypersensitive sites within the *β*-globin locus control region. These findings updated the conventional view that controlling gene expression with engineered transcription factor at sites other than promoters was limited [26].

The fusion domain could also be the transactivation domain VP64 or VPR, the DNA methyltransferase 3A (DNMT3A), or the DNA demethylase TET. The dCas9-VPR complex is an escalated version of dCas9-VP64, where VP64 was further fused by two other transcription factors p65 and Rta to increase the transactivation efficiency. Both of them have well been demonstrated to be reliable gene activation tools. The dCas9-VP64 complex could directly activate the silenced *Oct4* gene in both human and mouse cells [27]. By targeting the *Ngn2* and *Neurod1* genes, the dCas9-VPR complex was shown to be capable to aid induced pluripotent cell (iPSC) neuronal differentiation [28, 29]. The dCas9-DNMT3A complex was proved to be able to induce methylation at targeted CpG sites within multiple gene promoters. The highest methylation rate was estimated to be 50% [30]. The dCas9-TET complex has been shown to be able to rescue epigenetically silenced gene via inducing demethylation at the targeted region in a B2MtdTomato K-562 cell line. Transient expression of dCas9-TET and guide RNA together leads to a long-term reactivation effect highly specifically [31].

Efforts have been made to improve epigenome editing efficiency. The first strategy is incorporating SunTag into the dCas9-epieffector complex. SunTag is a repeating peptide array that can simultaneously bind with multiple copies of a certain protein. Several studies have highlighted the utility of the dCas9-SunTag system in improving transactivation robustness. For example, via recruiting multiple transactivation domains, the dCas9-SunTag complex has been proved to be able to augment a transactivation effect significantly compared to the conventional dCas9-single activator domain complex [32]. In another example, demethylation efficiency of the dCas9-SunTag-TET1 complex could reach as high as 90% in 4 out of 7 loci tested, resulting in 1.7- to 50-fold upregulation of targeted genes both in vitro and in vivo [33]. Moreover, SunTag could also be fused with the dCas9-DNMT3A complex to augment CpG methylation at targeted loci. Using the dCas9-SunTag-DNMT3A complex, researchers methylated a region of 4.5 kb residing the *Hoxa5* gene with fairly low off-target to on-target ratio [34]. The second strategy is the CRISPR-SAM system developed by Feng Zhang Lab. SAM represents synergistic activation mediator. In this system, the tetraloop and stem loop2 are two RNA loops protruding outside the Cas9-guide RNA complex. RNA aptamers are added onto the two loops to recruit more activation domains [35]. Similar to the dCas9-SunTag-epieffector strategy, the CRISPR-SAM system could also be used to recruit epieffector domains to improve the efficiency of epigenome editing. Another strategy is to optimize the system components, which may be of more uncertainty. For instance, in primary mouse T cells, researchers did not stabilize *Foxp3* expression significantly using the dCas9-TET1 complex, although demethylation at the targeted enhancer region was induced successfully, while switching to the dCas9-p300 complex sustained *Foxp3* expression even under inflammatory condition by inducing acetylation at the targeted promoter region [36].

**Figure 1:**
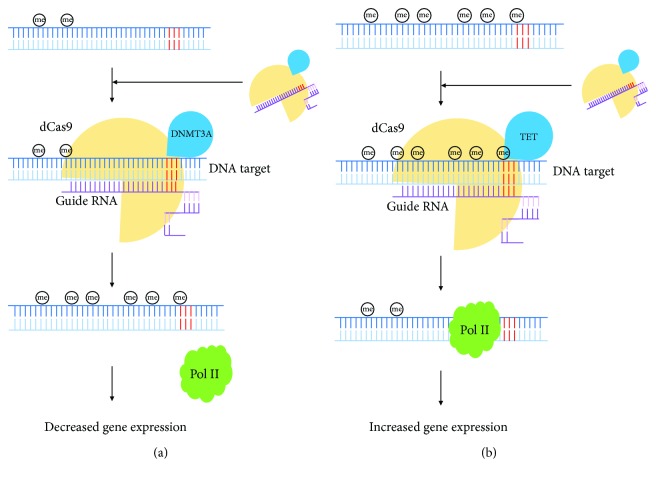
DNA methylation and demethylation mediated by Cas9. DNA sequences are represented by the blue lines. PAM sequences are highlighted by red color. Guide RNA sequences are shown in purple color. DNA polymerase II (PoI II) is represented by the green irregular shape. The circled “me” represents methylation at a specific CpG site. Basically, dCas9 functions as a DNA-binding protein. DNMT3A or TET is the epieffector. The dCas9-epieffector complex is guided to the DNA target by guide RNA via Watson-Crick base pairing to execute (a) DNA methylation or (b) demethylation, thus inducing decreased or increased gene expression.

**Figure 2:**
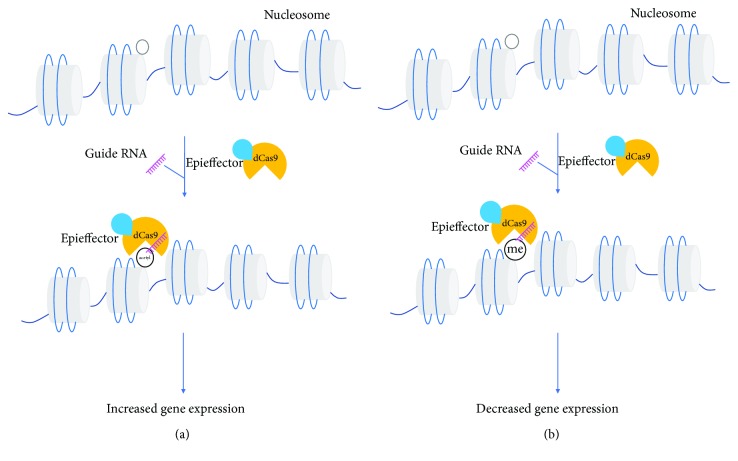
Histone acetylation and methylation mediated by Cas9. The empty circle attached to the nucleosome represents a specific amino acid of the histone side chain. The circled “acetyl” and “me” represents acetylation and methylation of amino acids, respectively. The dCas9-epieffector complex could be guided to a selected DNA target to achieve (a) acetylation or (b) methylation at histone level to regulate gene expression.

Briefly, the dCas9-epieffector complex could achieve methylation and demethylation at DNA level, rewriting histone marks by inducing methylation or acetylation at nucleosome level, and be optimized to improve the editing efficiency (Figures 1 and 2, Table 1).

### 1.2. Temporal and Spatial Control of Epieffectors

In a broad sense, epigenetics refers to the temporal and spatial control of gene expression, of which the total effect would determine cell phenotypes during development, aging, and disease pathogenesis [2, 37]. Thus, controlling gene expression with temporal and spatial precision is of great value. By inducing or repressing the expression of interested epigenetic regulators in iPSC models, differentiated cell lineages, and animal models, researchers may observe stage- and lineage-specific functions [14, 37, 38]. Generally, there are two inducing strategies, light and chemicals. Their combination with the CRISPR/Cas9 system is briefly summarized here.

Studies prior to the emergence of CRISPR have proved the utility of optogenetics in temporal control of gene expression. Basically, there need to be two components, a DNA-binding protein fusing to a light-sensitive cryptochrome protein, and a chromatin modifier fusing to a cryptochrome protein interaction partner, vice versa. [39, 40] When recruiting, the chromatin modifier to the targeted DNA sequence was controlled by blue light illumination, which would trigger conformation changes and rapid association between the cryptochrome protein and its interactor [1, 41, 42]. In the first example, the optically inducible dimerizing protein pair CRY2-CIB1 was fused to DNMT3A and telomere repeat binding factor-1 (TRF-1), respectively. The CRY2-DNMT3A fusion construct was the epigenetic rewriter, while the CIB1-TRF1 fusion construct was the target locator. Being shed with blue light, the CRY2-DNMT3A fusion construct would be targeted to the subtelomeric region to bind with the CIB1-TRF1 fusion construct and to execute locus-specific cytosine methylation. This system provided a method for understanding the role of subtelomeric methylation in telomere length maintenance [43]. The DNA-binding domain used in this experiment is TRF-1; it is reasonable to speculate that substitution of dCas9 with TRF-1 may achieve the same goal. Similarly, another study achieved site-specific epigenome editing at the *Ascl1* gene promoter using a CIB1-TALE fusion construct as the locus-specific binding complex. When DNMT3A catalytic domain was the rewriter, the *Ascl1* promoter was hypermethylated, resulting in decreased gene expression. When TET1 catalytic domain was the rewriter, the *Ascl1* promoter was hypomethylated, resulting in increased gene expression. By controlling the gene expression temporally, researchers were able to affect the differentiation preference of neural stem cells [44]. Moreover, the LITE system developed by Feng Zhang Lab was also a typical example of the optogenetic two hybrid systems. In this system, programmable TALE-DNA-binding domain was fused with CRY2 protein, while transcription activator domain VP64 or histone effector domain was fused with CIB1. Upon light stimulation, CRY2 would interact with CIB1, bringing effector domains to the genomic loci targeted by TALE to achieve transactivation or histone modification. With a suitable delivery method, this system could be applied both in vivo and in vitro [42].

By the same principle, the dCas9 DNA-binding domain paired with optical-inducible proteins could also be utilized to recruit the epieffector domain to the targeted DNA site in an inducible and reversible manner described above.

In a light-activated CRISPR/Cas9 effector (LACE) system, CRY2 and CIB1 were fused to transactivation domain VP64 and catalytically deactivated Cas9, respectively. Cotransfection of these fusion protein pairs with guide RNA resulted in detectable levels of gene activation in the presence of blue light. Interestingly, when the N-terminal fragment of CIB1 was fused to both N- and C-terminus of dCas9, gene activation level in response to blue light was significantly increased, which is consistent with previous observation that simultaneous recruitment of VP64 domains to the target site had a synergistic effect on gene activation [45].

Another similar strategy from a different angle is to split Cas9 into fragments and further tether them with optical inducible protein pairs. Upon light stimulation, Cas9 fragments would be brought together via light-induced dimerization to reconstruct nuclease activity. Interestingly, in a photoactivatable CRISPR/Cas9 system, when split Cas9 fragments were fused with the CRY2-CIB1 pair, no light-induced Cas9 activity was induced. Given the steric block effect may be at play, a different pair of proteins with much smaller molecular weight called magnets was used. The nuclease activity was successfully reconstructed upon light stimulation when Cas9 fragments were tethered with positive and negative magnets, respectively [46]. In addition to the CRY2-CIB1protein pair and magnets, the split Cas9 could also be bound with other ligand-binding protein pairs, which would be brought together by different chemicals, to achieve multiplexed genome or epigenome regulation simultaneously and rapidly [47, 48].

As to the chemical-controlled strategy, the optimized inducible gene knockdown or knockout (OptiKO) system was a good example. It was a strategy harnessing the CRISPR/Cas9 technology and tetracycline-inducible expression cassette in human iPSC. The OptiKO system has two main components. Cas9 was constitutively expressed while guide RNA (gRNA) was tetracycline-inducible. Clonal lines incorporating both the CAG-Cas9 cassette and the TET-ON/CAG-gRNA cassette was first isolated. In the following experiments, the addition of tetracycline to the isolated clonal cells at different time points could achieve targeted region knockdown or knockout flexibly. In particular, inducible knockdown of the *DPY30* gene, an epigenetic modifier, during the iPSC differentiation process revealed that *DPY30* not only was indispensable for germ layer specification but also played dynamic roles in iPSC differentiation and maturation of specific lineages, which exemplified the utility of OptiKO in an epigenetic study [49].

Although more and more evidence is being reported regarding the feasibility of optical or chemical controllable epigenome editing, the detailed mechanisms remain to be elusive. Whether the local chromosomal or nucleosome context affects the binding affinity of the DNA-binding protein remains to be determined. What are the exact effects of locus-specific epigenome editing on DNA epigenetic profiling, histone modifications, and chromosomal interactions? What are the transcription factors participating in transcription activation or repression mediated by epigenome editing? Do we have the confidence to ensure every shot with unfailing accuracy? Such questions need to be answered to achieve reliable epigenome editing consequence and bona fide conclusions.

**Figure 3:**
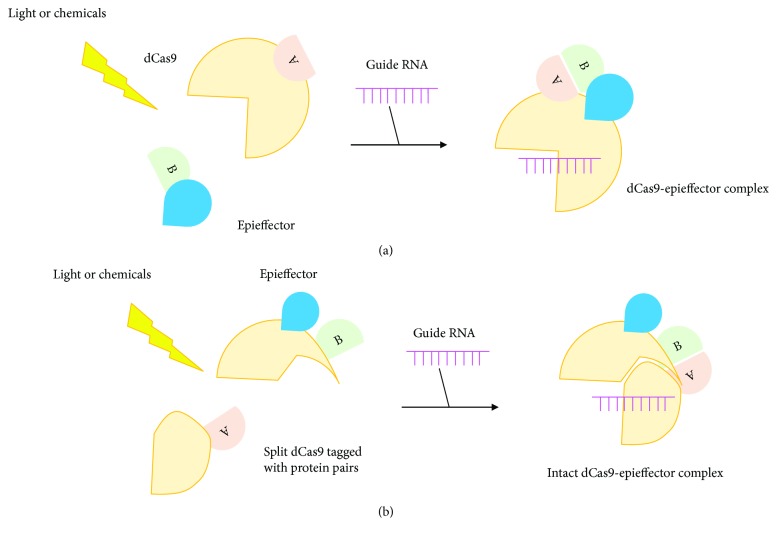
Schematic of temporal and spatial control of epigenome editing. The semicircles labeled with (A) and (B) represent a protein pair. The magenta comb-like lines represent guide RNA sequences. (A) is bound to dCas9, which would be directed by the guide RNA to the DNA target. (B) is bound to the epieffector. (a) Upon stimulation of light or chemicals, (A and B) would pair with each other thus bringing the dCas9 and epieffector together to achieve site-specific epigenome editing at a given time point. (b) Another strategy is splitting the dCas9 into two parts, each of which is bound by protein (A) or (B). Upon light or chemical stimulation, (A) and (B) would gather together to reconstruct an intact dCas9-epieffector complex to achieve site-specific epigenome editing at a given time point.

Elusive as the mechanism is, studies discussed above indeed enlightened us with new possible routes to perturb epigenome precisely. It is conceivable that through temporal and spatial control of genes playing indispensable roles in epigenetic dynamic changes, we may acquire new insights into the underlying mechanisms (Figure 3).

### 1.3. Noncoding RNA Manipulation

Noncoding RNA plays important roles in gene imprinting and chromatin remodeling; thus, it is an indispensable topic in the epigenetic research field [50, 51]. Accumulating evidence suggests that the CRISPR/Cas9 system is also a potential tool for studying noncoding RNAs [52, 53]. Firstly, CRISPR/Cas9 has been shown to be potential for manipulating noncoding RNA expression, including microRNA, long noncoding RNA, and miRNA families and clusters. For example, integrated Drosha and Dicer sites are essential for miRNA biogenesis. In a recent study, these two sites were used as the target cut sites of Cas9 nuclease in a cell model. The double-stranded breaks generated by Cas9 were further repaired by NHEJ mechanism. In this way, the integrity of the Drosha and Dicer sites was disturbed, which resulted in the downregulation of the corresponding miRNA and upregulation of genes regulated by them [54]. Another interesting example is using epigenome editing to aid a functional study of an imprinted gene, in which CRISPR/Cas9 was used to knock out a maternally expressed lncRNA gene, *Rian*, in mouse models. With a simultaneous delivery of multiple guide RNAs spanning the targeted region, the knockout rate could reach 33%. The *Rian*−/− mouse model facilitated functional studies of the gene knockout impact on nearby relevant gene expression in different tissues [55]. In addition, with multiple guide RNAs being delivered at the same time, CRISPR/Cas9 could be a scalable and multiplex system for miRNA family mutagenesis. The mutant rate could be as high as 90% [56]. Moreover, the CRISPR/Cas9 deletion-based method could be used to screen lncRNA involved in transcription regulation. In a recent research, researchers first generated a NF-kappa B reporter cell line constitutively expressing Cas9 nuclease. The activity of NF-Kappa cells was reflected by GFP signal via FACS. In the following steps, guide RNAs were delivered, along with Cas9, to achieve targeted lncRNA deletion. Changes in the GFP signal intensities and the levels of relevant proteins regulating or regulated by NF-kappa were further measured to determine whether the deleted lncRNA had a role in the interfered pathway. In this way, two lncRNAs, lincRNA-Cox2 and lincRNA-AK170409, were identified as potential negative regulators of NF-kappa expression in macrophage immune functions [57]. Conversely, noncoding RNA within cells could also be used to modulate Cas9 activity. A miRNA responsive CRISPR/Cas9 system was developed recently. Sequences complementary to a specific type of miRNA were incorporated into the 5′-UTR of the gene encoding Cas9, rendering the CRISPR/Cas9 system only switch-on in cells without such miRNA expression [58].

Most recently, Feng Zhang Lab developed a RNA editing tool, named as the RNA Editing for Programmable A to I Replacement (REPAIR) system. It was adapted from the type IV CRISPR/Cas13 system, where Cas13b was deactivated for its RNA cleavage capacity while the RNA-binding ability was reserved. Deactivated Cas13b was further fused with an adenosine deaminase enzyme called ADAR. After being directed to the targeted RNA transcript, the Cas13b-ADAR complex would deaminize adenosine to inosine, which is an analog to guanine in diverse biological processes, resulting in A to I conversion. Although this CRISPR/Cas13-based RNA editing tool seemed to have no overwhelming advantage over the CRISPR/Cas9-based DNA editing method in terms of specificity due to a substantial number of off-target events across the transcriptome, it still presented some promising merits. For example, no PAM sequence constraints within the RNA target meant more flexible target binding capacity of Cas13b compared to the classical Cas9. Direct conversion of RNA base without reliance on endogenous repair mechanism also rendered it applicable in mitotic cells such as neurons. Additionally, the transient nature of RNA editing may provide a basis for the temporal control of targeted RNA transcription and downstream translation [59].

Altogether, it seems that Cas9 instead of dCas9 is playing important roles in noncoding RNA manipulation, as more and more Cas proteins are being characterized, such as cpf1 [60, 61]. It may be likely that the design idea for CRISPR/Cas9 could also be expanded to other Cas protein types. We should keep learning from and trying to borrow wisdom from the world of microbes.

### 1.4. Characterization of Chromatin Structures and Interactions

DNA wraps around histones to form nucleosomes. Nucleosomes continue to assemble in a spiral pattern. Through several rounds of such repeated twisting, DNA and protein are finally packaged into the superhelix structure, chromatin. Chromatin has two statuses, the tightly packed heterochromatin and the loosely packed euchromatin [1, 62]. The former is inaccessible to transcription or translation, while the latter is transcriptionally or translationally active. Switch between these two conformations and chromatin-chromatin interactions play an important role in gene regulation [63]. The novel methods investigating chromatin structures and interactions presented by the emergence of the CRISPR/Cas9 technology are briefly summarized here.

**Figure 4:**
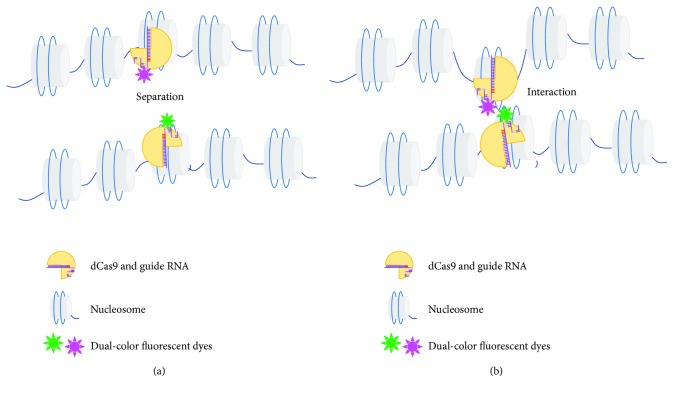
In vivo imaging of chromatin interaction mediated by Cas9. Guide RNAs direct dCas9 and fluorescent proteins to bind with selected DNA targets, forming the dCas9-gRNA-fluorescent dye complex. At chromosomal level, spatial position changes between chromosomal regions could be reflected by the (a) seperation or (b) interaction of the dual-color fluorescent signals.

Live cell imaging is a valuable method for studying structure changes and interactions of chromatin in epigenetic regulation. Efforts have been made to adapt the CRISPR/Cas9 system for the in vivo imaging experiments. Generally, the design rationale is tagging the guide RNA loops or the dCas9 protein with fluorescent dyes to form a dCas9-gRNA-fluorescent dye complex. In a recent study, a dCas9 expression cassette was first incorporated into cells of interest. Guide RNA scaffold was redesigned, with two new stem loops protruded. One of the loops was tethered by protein MS2; the other was bound by protein PP7, which were able to recruit EGFP-conjugated MCP and mCherry-conjugated PCP, respectively. Clonal cells expressing dCas9, EGFP, and mCherry were selected as the tested platform. The delivery of guide RNAs into these cells would bring dCas9 and fluorescent proteins together at targeted loci. Colocalization and separation of the two-color fluorescent signals indicated possible interactions between the targeted loci on chromosomal level. Using this dual-color system, researchers achieved real-time labeling of major satellites, minor satellites, and two single loci on chromosomes [64]. In another study, fluorescent proteins were tagged with dCas9 to achieve dual-color imaging in live human RPE cells. Researchers first isolated a cell line expressing dCas9-EGFP and dCas9-mCherry stably. Next guide RNAs were delivered to lead dCas9-fluorescent dye complex to the target, giving out observable fluorescent signals [65] (Figure 4). Intriguingly, based on similar design principles, an earlier study has explored the possibility of CRISPR/Cas9-mediated multicolor imaging in live human cells and successfully utilized this method to measure the distance between loci on the same or different chromosomes [66]. Moreover, in living cells, the CRISPR/Cas9 system has also been optimized for visualization of repetitive and nonrepetitive genomic sequences, to monitor telomere length dynamically and to track MUC4 locus variations in the mitosis process. Stably and inducible expression of dCas9-EGFP in the cell line being tested was a prerequisite. Delivering guide RNA into these cells at different stages would give out observable fluorescent signals, to some extent reflecting the length and conformational structure of the targeted genomic loci. Repetitive sequences need only one type of guide RNA, while nonrepetitive sequences need a bunch of guide RNAs spanning the target region to achieve enrichment of fluorescent signal at the targeted loci [67].

In addition to live cell imaging, CRISPR/Cas9 could also be used to characterize locus-specific regulatory composition. In a recent study, the deactivated cas9 nuclease was biotinylated to capture telomeric factors and components of enhancers regulating human *β*-globin gene expression. Chromatin interactions were isolated at high resolution, unbiased analysis of which suggested spatial features that may be involved in transcription regulation [68]. Another example is the engineered DNA-binding molecule-mediated chromatin immunoprecipitation (enChIP) technique, which could be comprehended as an adapted version of the conventional ChIP-seq method. dCas9 and guide RNA here were used to locate an interested genomic region. Next, cell lysis was crosslinked and sonicated to yield chromatin fragments, the dCas9-gRNA tagged of which was pulled down for further RNA-seq or mass spectrometry analysis [69, 70].

Chromatin structures and interactions are gaining increasing academic attention in recent years. It has been widely accepted that the dynamic chromatin changes play pivotal roles in the temporal-spatial regulation of genes. The in vivo imaging technique and several other attempts described above indicated that CRISPR/Cas9 is a promising research tool for this topic.

### 1.5. Epigenetic Element Screening

The accumulation of work has demonstrated the capacity of CRISPR/Cas9 for screening both coding and noncoding DNA sequences on a genome-wide scale [71]. Although the amount of evidence focusing on epigenetic screening approach is far less, efforts have been made to prove that CRISPR/Cas9-mediated epigenome editing is a potential method for enhancer screening and annotation [37]. For example, a recent study developed a CRISPR/Cas9-based epigenetic regulatory element screening system, abbreviated as CERES, for regulatory element annotation in the native chromosomal background. Firstly, cells constitutively expressing dCas9-KRAB repressor or dCas9-p300 activator were, respectively, transduced with gRNA libraries targeting regions containing putative regulatory elements surrounding the gene of interest for loss-of-function or gain-of-function screens. Next, given the interested gene was a transmembrane protein, FACS was used to sort cells for increased and decreased gene expression. Finally, guide RNA enrichment analysis was used to identify functional enhancers [72]. Albeit publications on this topic are being few at present, it is still believable that the CRISPR/Cas9-mediated epigenome editing has the potential to pave the way for studying putative regulatory elements in their native chromosomal background in a high-throughput manner.

### 1.6. Potential Clinical Applications

By transcriptionally activating or deactivating a specific gene that is normally silent or active, epigenome editing exhibited therapeutic potentials. It is easy to imagine that diseases with aberrant epigenetic marks as the underlying pathophysiologic mechanisms would be excellent targets to test epigenome editing-based therapeutics. The disease spectrum may include cancers, neurodegenerative diseases, neuropsychiatric diseases, neurodevelopmental diseases, and imprinting disorders. For example, using a dCas9-SAM-VPR complex, researchers reactivated a heavily methylated tumor suppressor gene, *Maspin*, in lung cancer cells both transcriptionally and translationally, which limited cancer cell growth and apoptosis [73]. Another interesting example is that the delivery of ZFN-VP64 complex into mouse models of Alzheimer's disease could rescue the memory deficit phenotype by upregulating a major synaptic protein PSD95 [74]. Although the DNA-binding domain used in this study was ZFN, it still validated the possibility of dCas9-mediated epigenome editing for treating neurodegenerative disorders to some extent. As to the neurodevelopmental diseases, epigenome editing theoretically is a potential therapeutic strategy worth a try, especially for diseases caused by aberrant imprinting patterns without underlying genetic mutations, such as the Angelman syndrome, where hypomethylated imprinting center of the maternal chromosome led to epigenetic silencing of the *Ube3a* gene on both alleles [75]. With a suitable dCas9-epieffector complex, the imprinting center may be remethylated to correct the abnormal imprinting pattern and thus reactivate the *Ube3a* gene epigenetically. Other representative candidates may include the Rett syndrome, Huntington's disease, and Friedreich ataxia, where dCas9-epieffector complex may be harnessed to restore or repress the responsible gene to cure the disease [12, 76, 77].

In addition, epigenome editing has the potential to facilitate stem cell therapy. In recent years, iPSCs have been a major source for cell replacement therapy due to its accessibility and pluripotency [78–82]. iPSCs experience active and delicate epigenome reset to become differentiated cells, and vice versa [83]. By changing the epigenome landscape at a desired time point or cell stage, epigenome editing may be utilized to improve the reprogramming efficiency or to confine the differentiation direction to yield more pure target cell populations for cell replacement therapy [84, 85]. Moreover, epigenome editing provides a new route for the transdifferentiation process. For example, using the CRISPR-SAM system to target and activate pdx1, a gene essential to pancreatic development, researchers successfully transdifferentiate liver cells into insulin-producing pancreatic cells to treat diabetes mellitus in mouse models [86]. Last but not least, epigenome editing holds a great promise for the in vivo reprogramming process. In vivo overexpression of the Yamanaka factors have proven to be able to fully or partially help somatic cells to regain pluripotency in situ. These rejuvenated cells would subsequently differentiate again to replace the lost cell types [87–89]. Since the reprogramming process essentially is the reset of epigenome, it is fair to envision that epigenome editing designed to reset the soma epigenome to a naïve state may be a more controllable and precise method for in vivo reprogramming.

Taken together, these studies demonstrated the therapeutic potential of CRISPR/Cas9-mediated epigenome editing in various diseases and stem cell therapy.

## 2. Concluding Remarks and Future Perspectives

In conclusion, all of the studies described above indicated that CRISPR/Cas9-mediated epigenome editing holds a great promise for epigenetic studies and therapeutics. However, there are still some limitations to be scrutinized. First of all, in terms of basic science studies, although most studies claimed high specificity in their experiments, however, the high specificity usually is the result of repeated optimization. A precise model that could predict deleterious off-target effects during the experiment design stage is still lacking. In addition, although transactivation or repression effects on multiple genes were well documented in publications, mechanisms underlying the phenomenon were not clear. Epigenetic mark profiling on epigenome scale was not sufficient. Local CHIP-seq data usually only focused on the characterization of one or few histone marks. Theoretically, we hope that epigenome editing could achieve targeted gene regulation by changing epigenetic marks specifically and freely according to our wills. To achieve this goal, high specificity and clarified mechanisms are the prerequisite. Therefore, more thorough off-target event assessments and more studies focusing on mechanisms underlying epigenome editing are needed.

Moreover, in terms of clinical applications, several issues need to be addressed prior to successful clinical translation. Firstly, the endurance of gene activation or a repression effect mediated by CRISPR/Cas9 remains to be undetermined. It has been thought that epigenome-editing-induced gene activation or repression is short-term [90, 91]. On the contrary, there was also evidence showing that a gene silencing effect mediated by the hit-and-run epigenome editing strategy could also be long-term and inheritable [31]. A short-term effect is more suitable for antagonizing acute pathogenic factor exposure and the transdifferentiation process. However, for treating chronic diseases, a long-term effect is expected. Additionally, a safer and efficient delivery method should be developed [92]. Adeno-associated virus (AAV) vectors have been the prevailing delivery method for some time. By tagging a synthetic surface peptide, splitting the Cas9 protein or using its smaller orthologues, and choosing a suitable administration route, researchers significantly improved the packaging capacity and delivery efficiency of AAV vectors [93–96]. However, for clinical applications, more optimization is required. The immunogenicity of AAV vectors, dCas9 proteins, and guide RNAs should be determined precisely. The off-target effects of AAV vectors or dCas9-epieffector complex should be minimized as much as possible to ensure clinical safety [97].

## Figures and Tables

**Table 1:** Validated representative dCas9-epieffector complex.

Type	Mechanism	Function	Reference
dCas9-KRAB	Histone methylation	Gene downregulation	[21, 27]
dCas9-DNMT3A	DNA methylation		

dCas9-LSD1	Histone acetylation	Gene upregulation	[22–26, 28]
dCas9-p300	Histone acetylation		
dCas9-TET	DNA demethylation		
dCas9-VP64 or VPR	Recruitment of active transcription factors		

dCas9-SunTag-epieffector	Recruitment of multiple epieffector domains by the repeating peptide array SunTag	Effect augmentation	[29–32]
dCas9-SAM-epieffector	Recruitment of multiple epieffector domains by the protruding guide RNA loops		
